# The Effect of Peritoneal Air Exposure on Intestinal Mucosal Barrier

**DOI:** 10.1155/2014/674875

**Published:** 2014-08-20

**Authors:** Jun Bao, Shanjun Tan, Wenkui Yu, Zhiliang Lin, Yi Dong, Qiyi Chen, Jialiang Shi, Kaipeng Duan, Xiaowu Bai, Lin Xu, Jieshou Li, Ning Li

**Affiliations:** ^1^Department of General Internal Medicine, Jiangsu Province Official Hospital, Nanjing 210024, China; ^2^Research Institute of General Surgery, Jinling Hospital, Medical School of Nanjing University, Nanjing 210002, China; ^3^Research Institute of General Surgery, Jinling Hospital, Clinical School of Nanjing, Second Military Medical University, Nanjing 210002, China

## Abstract

*Background*. Damage of the intestinal mucosa barrier may result in intestinal bacterial and endotoxin translocation, leading to local and systemic inflammation. The present study was designed to investigate whether peritoneal air exposure induces damage of intestinal mucosal barrier. *Methods*. Sprague-Dawley rats (weighing 210 to 230 g) were randomized into five groups (6/group): a control group, a sham group, and three exposure groups with peritoneal air exposure for 1, 2, and 3 h, respectively. At 24 h after surgery, blood and terminal ileum were sampled. The serum D-lactate levels were determined using an ELISA kit. The intestinal permeability was determined by measuring the intestinal clearance of FITC-dextran (FD4). The histopathological changes in terminal ileum were also assessed. *Results*. Compared with the controls, peritoneal air exposure caused an increase in both serum D-lactate level and intestinal FD4 clearance, which were proportional to the length of peritoneal air exposure and correlated to Chiu's scores, indices for intestinal mucosal injury. Edema and inflammatory cells were also observed in mucosa and submucosa of ileum in three exposure groups. *Conclusions*. Peritoneal air exposure could induce damage to the intestinal mucosal barrier, which is proportional to the time length of peritoneal air exposure.

## 1. Introduction

Peritoneal air exposure is a common clinical phenomenon in open abdominal surgery. Although it is well accepted that peritoneal air exposure induces injury to various tissues/organs, the etiology and underlying mechanisms are not fully understood. It is reported that peritoneal air exposure induces intestinal and systemic inflammatory response [1, 2]. However, the originating site of inflammation after peritoneal air exposure is unknown; it is also unclear whether the inflammatory response is related to the duration of peritoneal air exposure.

The intestinal tract is one of the target organs during various stresses [3]. Damage to the intestinal mucosa barrier will result in intestinal bacterial and endotoxin translocation and further contribute to local and systemic inflammations [4]. In clinical practice, patients with serious gastrointestinal failure undergoing open abdominal surgery often show systemic inflammatory syndrome. Therefore, the injury and inflammation in the intestinal tract are often considered as the driving force of multiple organ dysfunction syndrome (MODS). Investigations to explore the risk factors for damage to intestinal mucosal barrier and to develop the intervention approaches to restoring the barrier function are popular pursuits in modern surgical research.

The present study was designed to investigate whether peritoneal air exposure leads to intestinal mucosal barrier damage and whether this effect is related to the duration of peritoneal air exposure under experimental conditions mimicking clinical operation room atmosphere.

## 2. Materials and Methods

### 2.1. Animals

Healthy adult male Sprague-Dawley rats (weighing 210 to 230 g) were obtained from Jinling Hospital, Nanjing, China. The rats were housed in our laboratory in a temperature- and humidity-controlled environment; they had free access to standard rat chow and tap water. The lights were maintained on a 12 : 12 h light : dark cycle. The animals were allowed a minimum of one week acclimatization before experiments. This animal use and care protocol and experimental procedures were reviewed and approved by the Institutional Animal Care and Use Committee of Jinling Hospital. The experiments were performed according to the National Institutes of Health Guidelines on the use of laboratory animals.

### 2.2. Experimental Procedures

Thirty rats were randomly divided into five groups (*n* = 6 each): a control group, a sham group, and three exposure groups with peritoneal air exposure for 1, 2, and 3 h, respectively. The animals were anesthetized by subcutaneous injection of 2% pentobarbital sodium (3.5 mL/kg body weight) [5], and the total anesthetic time was 4 h for all the five groups of animals. The surgical procedures were performed in an aseptic environment with controlled temperature and humidity. For the exposure groups, after the anesthesia was induced, a 3 cm midline abdominal incision was made, and then the wound edge was retracted to allow for maximal peritoneal air exposure for 1, 2, and 3 h, respectively [1]. Thereafter, the abdomen was closed in one layer with 3–0 silk continuous suture. For the sham group, the animals underwent the same anesthesia operation procedure but without laparotomy. For the control group, the animals underwent the same anesthesia but without any operative procedures. The animals in all the five groups were monitored until they recovered from anesthesia and then returned to their corresponding cages. At 24 h after surgery, samples of blood and intestinal tissues were obtained after the animals were anesthetized. The selection of terminal ileum as the investigation site was based on previous publications [6–8]. These studies have indicated that the terminal ileum is the major site for observation of damage to gut barrier functions following exposure to various injuries. It is suggested that terminal ileum is the most sensitive section of the intestinal tract [9].

### 2.3. D-Lactate (D-LA) Determination

D-lactate is one of the major products of bacterial metabolism, which is hardly seen in normal circulation or body fluids. When the permeability of the intestine is increased after bacterial infection, the level of D-lactate will increase, penetrate across intestinal barrier, and enter the circulation, resulting in an increased blood level of D-lactate. Therefore, in the present study, D-lactate was chosen as a marker for the changes in intestinal barriers after peritoneal air exposure. The blood samples were obtained from the inferior vena cava. The serum was prepared by centrifugation at a speed of 1,500 rpm for 15 min at 4°C,  and  the  levels  of  D-LA were determined with an ELISA kit for rats (R&D Systems, Germany) according to the manufacturer's instructions.

### 2.4. Intestinal Permeability Determination

The intestinal permeability was also determined by calculating intestinal clearance of fluorescein-isothiocyanate dextran (FD4) as described in the literature [9, 10]. Briefly, a segment of terminal ileum with length of 8 cm was prepared, and the mucosa was everted. One end of the gut segment was ligated, and a gut sac was made by injecting 1.5 mL of Krebs-Henseleit bicarbonate buffer from the other end. The sac was then suspended in a solution containing 0.5 mg/mL of FD4, and the temperature was maintained at 37°C. The bathing solution was aerated by gently bubbling with a gas mixture containing 5% CO_2_ and 95% O_2_. Thirty minutes later, the value of the mucosal surface area (*A*) in the sac solution was measured by fluorescence spectrophotometer. The clearance of FD4 was calculated according to the following formula:

(1) C=[FD4]ser×1 mL[FD4]muc×30 min,FD4=5.0125×A−7.957, A=πLD,

where *C* is the mucosal to serosal clearance of FD4 in *μ*L · min⁡^−1^ · cm^−2^;  [FD4]ser is the FD4 concentration in the serosal fluid aspirated from the sac at the end of the 30 min period; [FD4]muc is the FD4 concentration in the serosal fluid aspirated from the sac at the beginning of the 30 min period; *L* is the length of the sac and *D* is the diameter of the sac.

### 2.5. Histopathology

The terminal ileum was fixed in 4% buffered formaldehyde and embedded in paraffin. Slices of 4 *μ*m thickness were prepared, stained with hematoxylin and eosin (H&E), and then examined by a pathologist blinded to this study design using light microscopy. The degree of intestinal mucosa injury was assessed by using Chiu's scoring system as described previously [11]. The intestinal mucosal changes after peritoneal air exposure were graded as follows: Grade 0, normal mucosal villi; Grade 1, development of subepithelial Gruenhagen's space, usually seen at the apex of the villus, often with capillary congestion; Grade 2, extension of the subepithelial Gruenhagen's space with moderate lifting of epithelial layer from the lamina propria; Grade 3, massive epithelial lifting down the sides of villi, with a few tips being denuded; Grade 4; denuded villi with lamina propria and dilated capillaries exposed, increased cellularity of lamina propria; and Grade 5, digestion and disintegration of lamina propria, hemorrhage, and ulceration. The average scores of the exposure groups were compared with that of the controls.

### 2.6. Statistical Analysis

Data were expressed as mean ± SD. Statistical analyses were performed using SPSS 17.0 software (SPSS Inc., USA). Data were analyzed using one-way analysis of variance (ANOVA) after homogeneity test for variance. Significant results were then analyzed post hoc using the Dunnett test. The correlation of two variables was performed using Pearson correlation analysis. Differences were considered statistically significant when  *P* < 0.05. The exact *P* values were presented.

## 3. Results

### 3.1. General Observations

All rats survived the entire protocol. There were no significant differences in body weight, normal behavior, and postoperative diet and fluid intake among the experimental groups and controls.

### 3.2. D-LA Level in Blood

**Figure 1:**
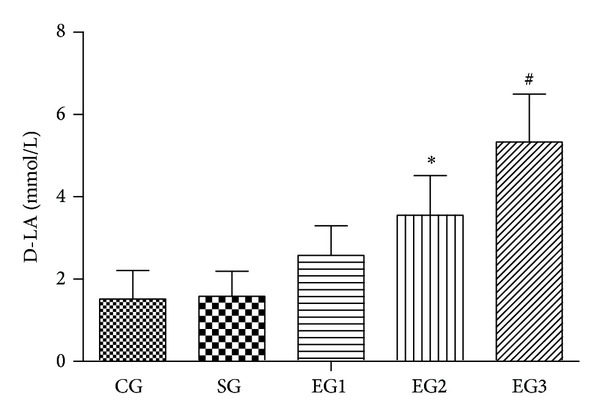
The level of D-lactate in each group. CG = control group; SG = sham group; EG1 = exposure group with peritoneal air exposure for 1 h; EG2 = exposure group with peritoneal air exposure for 2 h; EG3 = exposure group with peritoneal air exposure for 3 h. Values are expressed as mean ± SD. *P = 0.001, ^#^ *P* = 0.000, compared with CG.

As shown in Figure 1, there were no significant differences in serum D-LA levels between the control and sham group (*P* = 1.000). Peritoneal air exposure induced a progressive increase in the D-LA level, which differed significantly in the 2 and 3 h exposure groups, when compared with the control group (*P* = 0.001, *P* = 0.000, resp.).

### 3.3. Intestinal Permeability

**Figure 2:**
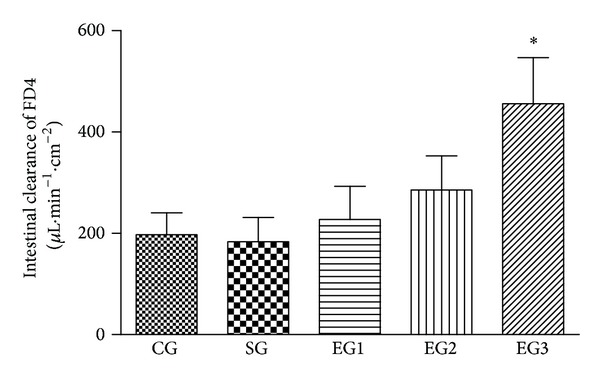
The intestinal clearance of FD4 in each group. CG = control group; SG = sham group; EG1 = exposure group with peritoneal air exposure for 1 h; EG2 = exposure group with peritoneal air exposure for 2 h; EG3 = exposure group with peritoneal air exposure for 3 h. Values are expressed as mean ± SD. *P = 0.000, compared with CG.

As shown in Figure 2, there were no significant differences in the intestinal clearance of FD4 between the control and sham group (*P* = 0.987). Peritoneal air exposure elicited a progressive increase in intestinal clearance of FD4, which differed significantly in the 3 h exposure group, when compared with the control group (*P* = 0.000).

### 3.4. Histopathology

**Figure 3:**
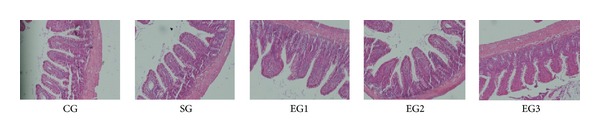
Histopathologic changes in the tissue of ileum using H&E staining and microscopy (×100). CG = control group; SG = sham group; EG1 = exposure group with peritoneal air exposure for 1 h; EG2 = exposure group with peritoneal air exposure for 2 h; EG3 = exposure group with peritoneal air exposure for 3 h.

**Figure 4:**
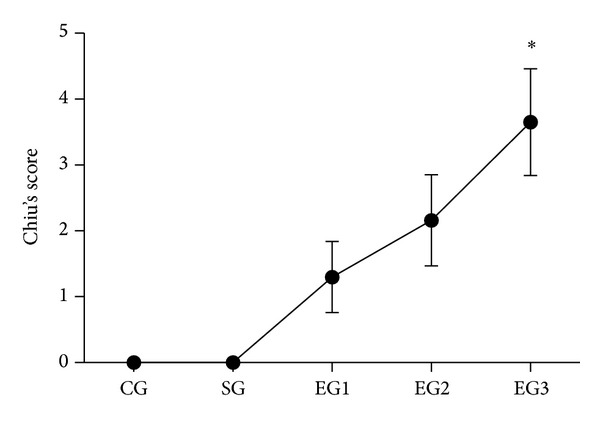
Chiu's score in each group. CG = control group; SG = sham group; EG1 = exposure group with peritoneal air exposure for 1 h; EG2 = exposure group with peritoneal air exposure for 2 h; EG3 = exposure group with peritoneal air exposure for 3 h. Values are expressed as mean ± SD. *P = 0.000, compared with EG1.

As shown in Figure 3, there was no obvious structural injury in ileum tissue among groups. However, edema and inflammatory cells were observed in the mucosa and submucosa of ileum in all the three exposure groups when compared with those of the control group. In addition, as shown in Figure 4, peritoneal air exposure elicited a progressive increase in Chiu's score, which differed significantly in the 3 h exposure group, when compared with the 1 h exposure group (*P* = 0.000).

### 3.5. Correlation Analysis

**Figure 5:**
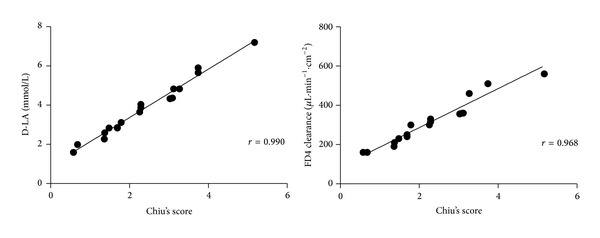
The analysis of correlation between D-lactate levels and Chiu's scores, and the correlation between the values of intestinal clearance of FD4 and Chiu's scores, respectively (*P* = 0.000).

As shown in Figure 5, both the serum D-lactate levels and the intestinal clearance rates of FD4 were positively correlated to Chiu's score with correlation coefficients of 0.990 and 0.968, respectively (*P* = 0.000).

## 4. Discussion

In the present study, we investigated whether peritoneal air exposure led to intestinal mucosa barrier damage and whether the effects were related to the duration of peritoneal air exposure. Our results showed that peritoneal air exposure caused increases in both serum D-lactate level and intestinal clearance of FD4, when compared with the control group. This difference was proportional to the exposure time length of peritoneal air exposure. In addition, edema and inflammatory cells were also observed in the mucosa and submucosa of ileum, although there was no substantial injury in the three exposure groups. Furthermore, both serum D-lactate level and intestinal clearance of FD4 were positively correlated to Chiu's score for intestinal mucosal injury.

Peritoneal air exposure is a common clinical phenomenon in open abdominal surgery impacting the patient's recovery after operation and quality of life. There are limited studies linking the severity of injury with peritoneal air exposure. Peritoneal air exposure could induce intestinal and systemic inflammatory response [1, 2]. For example, compared with the control group, the level of systemic inflammatory response was much greater in the experimental group when gut was exposed to air for one minute in rat induced by opening abdomen with 2 cm incision [2]. However, what location this inflammation originates from during peritoneal air exposure is still unclear.

Bacterial colonization of the gut is extensive and bacteria are confined to the gastrointestinal tract by the mucosal barrier. Damage to the intestinal mucosa barrier will result in intestinal bacteria and endotoxin translocation and further contribute to local and systemic inflammations [4]. Patients with serious gastrointestinal failure undergoing open abdominal surgery often have systemic inflammatory syndrome. Therefore, in order to elucidate the mechanism of the systemic inflammation induced by peritoneal air exposure, it is important to determinate the change in intestinal mucosa barrier during peritoneal air exposure.

It is a challenging task to measure the integrity and/or function of the intestinal mucosal barrier. In many published reports, measuring the intestinal permeability is employed to indirectly assess the barrier function. For example, D-lactate has been used as a biomarker for the permeability of the intestines, especially for bacterial infection [12–15]. Therefore, we choose D-lactate as a marker for the changes in intestinal barriers after peritoneal air exposure. In addition, FD4 is a relatively large molecule, which could not penetrate the normal intestinal barrier. When the intestinal permeability is increased under pathological conditions, FD4 can come across the intestinal barrier. Therefore, the FD4 clearance is also a good marker for function of intestinal barrier [9, 10]. In the present study, we found that peritoneal air exposure caused an increase in both serum D-lactate level and intestinal clearance of FD4. These changes were proportional to length of peritoneal air exposure. In addition, both serum D-lactate level and intestinal clearance of FD4 were positively correlated to Chiu's scores for intestinal mucosal injury. All of these results indicated that peritoneal air exposure could induce damage of intestinal mucosal barrier then cause an intestinal bacteria and endotoxin translocation and further contribute to local and systemic inflammation.

It is well known that patients undergoing longer length of operation time will have a higher level of surgical stress and suffer from more complications after surgery. According to our results in the present study, the time length of peritoneal air exposure may be also an important role in the development and progression of intestinal mucosal barrier damage. Therefore, in clinical practice, when patients undergo excessive abdominal surgery, methods to prevent peritoneal air exposure or reduce operation time may be an effective way to alleviate postoperative intestinal mucosal barrier damage, decrease systemic inflammatory response, and further enhance recovery after surgery.

Of note, there were some limitations in the present study. It has been well documented that several factors affect the development and progress of systemic inflammatory response syndrome after open surgery, including operation procedure and time, temperature, humidity, air atmosphere, and blood loss [16–18]. The present study attempted to mimic the clinical operation conditions and observed the relationship between air exposure time and the changes in intestinal barriers. Under our experimental conditions, the contribution of each individual factor cannot be clearly defined. Future studies are needed to investigate these factors in a better designed and well-controlled study setting. In the present study, the terminal ileum was chosen as the investigation site based on previous publications in the research field [6–8]. It is suggested that terminal ileum is the most sensitive section [9]. However, we could not completely rule out the possible effects of the bacterial translocation on other sections of the intestine such as colon. Future studies need to investigate other sections in addition to terminal ileum.

In conclusion, our results suggest that peritoneal air exposure could induce damage to intestinal mucosal barrier, and this effect is proportional to the time length of peritoneal air exposure. Every effort should be made to prevent prolonged peritoneal air exposure during surgery.
